# Prognostic Impact of Inflammatory Markers PLR, LMR, PDW, MPV in Medullary Thyroid Carcinoma

**DOI:** 10.3389/fendo.2022.861869

**Published:** 2022-03-08

**Authors:** Canxiao Li, Han Zhang, Shijie Li, Daqi Zhang, Jingting Li, Gianlorenzo Dionigi, Nan Liang, Hui Sun

**Affiliations:** ^1^Division of Thyroid Surgery, The China-Japan Union Hospital of Jilin University, Jilin Provincial Key Laboratory of Surgical Translational Medicine, Jilin Provincial Precision Medicine Laboratory of Molecular Biology and Translational Medicine on Differentiated Thyroid Carcinoma, Changchun, China; ^2^Division of General and Endocrine Surgery, Istituto Auxologico Italiano Istituto di Ricovero e Cura a Carattere Scientifico (IRCCS), Department of Medical Biotechnology and Translational Medicine, University of Milan, Milan, Italy

**Keywords:** inflammation-based score, medullary thyroid carcinoma, clinicopathological characteristics, calcitonin, biomarkers

## Abstract

**Background:**

Neutrophil-to-lymphocyte ratio (NLR), platelet-to-lymphocyte ratio (PLR), lymphocyte-to-monocyte ratio (LMR), mean platelet volume (MPV), and platelet distribution width (PDW) have been used as prognostic biomarkers in various cancers. We aim to investigate the relationship between the above inflammatory indices, clinicopathological features, and postoperative calcitonin (Ctn) progression in medullary thyroid carcinoma (MTC).

**Methods:**

Sixty-eight patients diagnosed with MTC who underwent surgery at our institution between 2009 and 2020 were retrospectively evaluated. Areas under the receiver operating characteristic curves (ROC) and logistic regression were applied to explore the potential risk factors.

**Results:**

PDW was predictive of lymph node metastasis (LN) (AUC=0.645, *P*=0.044), PLR, PDW, and MPV were predictive of capsule invasion (AUC=0.771, *P*=0.045; AUC=0.857, *P*=0.008; and AUC =0.914, *P*=0.002, respectively), and MPV and LMR were predictive of postoperative Ctn progression (AUC=0.728, *P*=0.003; AUC=0.657, *P*=0.040). Multivariate analysis revealed that PDW ≤ 16.4 [(OR=7.8, 95% CI: 1.532-39.720, *P*=0.013)] and largest tumor size ≥1 cm (OR=4.833, 95% CI: 1.514-15.427, *P*=0.008) were potential independent risk factors for lateral LN metastasis. We also found that, MPV ≤ 8.2(OR=13.999, 95% CI: 2.842-68.965, *P*=0.001), LMR ≤ 4.7 (OR=4.790, 95% CI: 1.034-22.187, *P*=0.045), and N1 (OR=45.890, 95%CI:3.879-542.936, *P*=0.002) were potential independent risk factors for postoperative Ctn progression. In addition, compared with the single indicator, the appropriate combination of MPV and LMR could improve the specificity and sensitivity of predicting postoperative Ctn progression.

**Conclusions:**

PLR, LMR, PDW, and MPV were associated with clinicopathological features and postoperative Ctn progression in MTC, suggesting that those inflammatory indices might be potential biomarkers of MTC.

## Introduction

Cancer-related inflammation plays a central role in the development and progression of malignancies (1). Specifically, In particular, an increasing number of studies have demonstrated the importance of host systemic inflammatory responses in clinical performance and tumor prognosis, and various inflammatory indices, including neutrophil-to-lymphocyte ratio (NLR), platelet-to-lymphocyte ratio (PLR), and lymphocyte-to-monocyte ratio (LMR), are now widely recognized as novel potential biomarkers for predicting cancer outcomes (2–4). Moreover, all inflammatory indices can be obtained as part of a routine complete blood count prior to surgery, so they do not impose an additional burden on patients. This suggests that an inflammation index has good applications in tumors.

Medullary thyroid carcinoma (MTC) originates from the parafollicular C-cells of the thyroid gland, which are derived from the neural crest (5). Although MTC accounts for only 1–2% of thyroid cancers, it is responsible for approximately 13% of all thyroid cancer-related deaths (6). However, there is still a lack of valid biomarkers to evaluate the clinicopathological features and prognosis of MTC. Given the potential of the aforementioned inflammatory indices, we aimed to investigate their potential applications in MTC.

Current American Thyroid Association guidelines recommend measuring serum calcitonin (Ctn) levels in patients diagnosed with histological MTC (5). However, the prognostic value of inflammatory index for postoperative Ctn progression has not been studied in MTC. Therefore, another aim of the present study was to clarify the value of inflammatory indices for postoperative progression of Ctn in MTC.

## Materials and Methods

### Design of the Study

Retrospective observational study

### Setting

China-Japan Union Hospital of Jilin College, China

### Time Frame

2009–2020

### Eligibility

The inclusion criteria were as follows: consecutive enrollment and first-time diagnosis of primary MTC, and confirmation of diagnosis by histological analysis and imaging techniques. The following patients were excluded: Patients who had previously received antitumor treatment, including chemotherapy, radiotherapy, and immunotherapy; patients with other primary cancers; follow-up for < 6 months; coinfection or other inflammatory diseases (except autoimmune thyroid diseases such as chronic lymphocytic thyroiditis), not resectable at the time of referral, and lack of relevant information. All patients who met the inclusion criteria during the study period were included in the study. In addition, follow-up was performed, and clinical features and outcomes were assessed. No intervention was performed during the treatment period

### Patients and Data Collection

**Figure 1:**
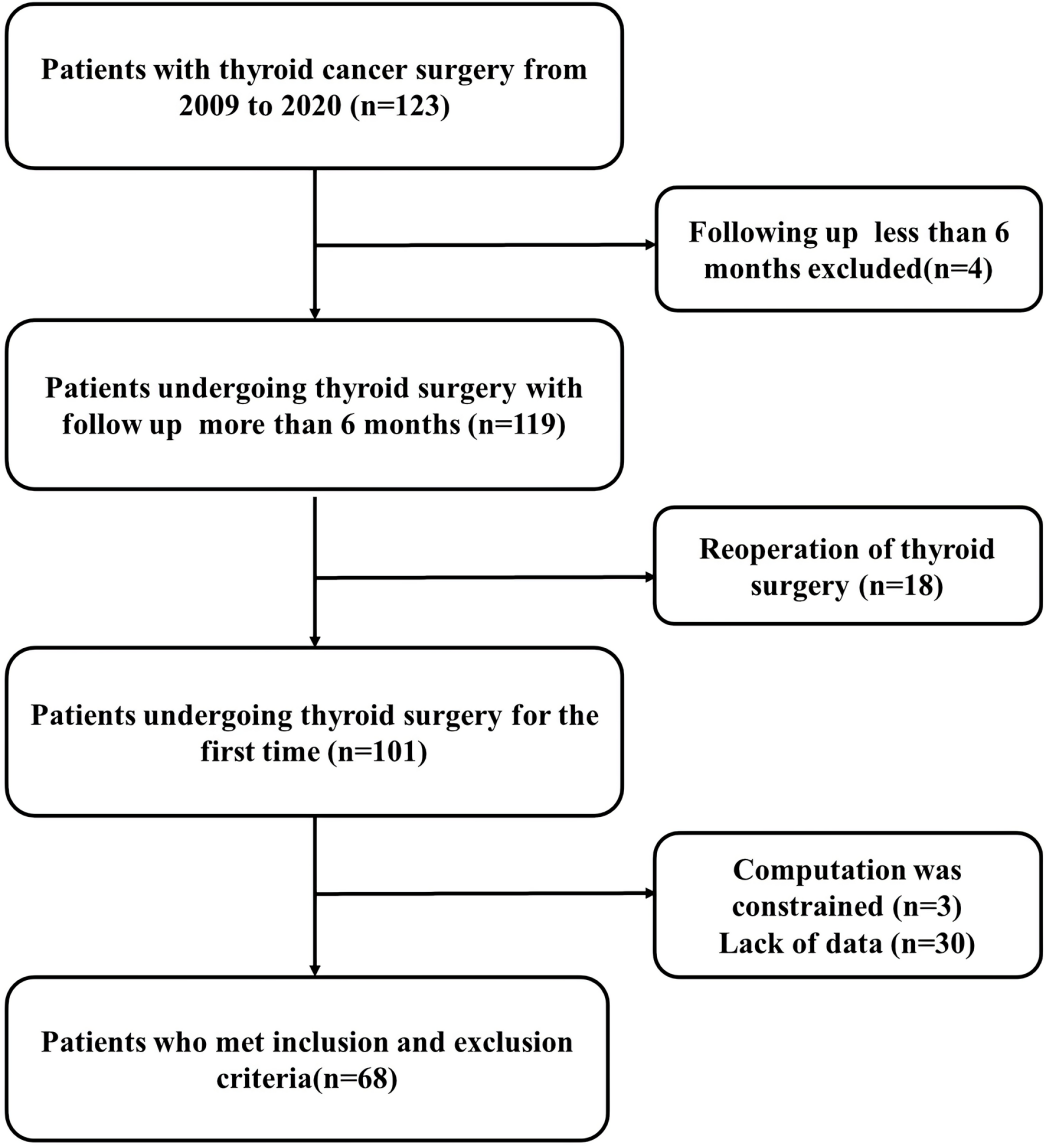
Flow diagram of the patients included in the current study.

A total of 101 patients had an initial diagnosis of MTC. Sixty-eight patients had complete data and follow-up, including preoperative complete blood count, Ctn serum levels, and clinical and histopathological data (**Figure 1**).

### Ethics

This study was approved by the institutional ethics committee of the China-Japan Union Hospital of Ji Lin College. Written informed consent was obtained from all the patients.

### MTC Diagnosis

Tumor tissue samples were obtained from all patients for histological analysis or surgical resection. MTC diagnosis was based on guidelines (7). Tumor size corresponded to the largest tumor diameter measured on pathological examination after surgery.

### Measurement of Biomarkers

NLR is the ratio of neutrophil count to lymphocyte count, PLR is the ratio of platelet count to lymphocyte count, and LMR is the ratio of lymphocyte count to monocyte count. The above inflammatory indices were calculated based on the preoperative complete blood count; Ctn was detected by chemiluminescence: Ctn (REF: LKCL1) using the Immulite 1000 system (Siemens Medical Diagnostics Solutions). The normal range of Ctn levels was 0.15−6.00 pg/mL in females and 0.15–9.20 pg/mL in males. The upper detection limit was 585.0 pg/mL.

### Follow-Up

By December 30, 2020, the follow-up time was 8–133 months, and the median follow-up time was 79 months.

### Outcomes Recorded

Considering the different tumor load of different patients, the assessments time of postoperative ctn can be divided into 1 week, 1 month, 3 months and half a year. If it is lower than the lower limit of detection or within the normal reference range, regular postoperative reexamination is adopted. The initial reexamination period is half a year, and if the condition is stable, it is gradually extended to once a year.

Patients were divided into three groups based on postoperative serum Ctn levels (5, 8). *Remission*: Postoperative Ctn levels decreased to normal and remained constant. *Stable*: Postoperative Ctn levels were stable but did not decrease to normal values. *Progression*: Postoperative Ctn level increased to 150 pg/mL or doubling time was <12 months (**Supplementary Table 1**).

### Statistical Analysis

The SPSS package version 23.0 (SPSS) was used to analyze all data. All results are presented as mean ± standard deviation (SD), median, and interquartile range (IQR) or number (%). Fisher’s exact test and rank sum test were used to analyze categorical variables. Continuous variables were analyzed using an ANOVA test or, in the case of non-normal distributions, the Kruskal-Wallis test. Binary logistic regression models were constructed to predict MTC, and the factors that contributed to the outcome in univariate analysis had a *P* < 0.05. and statistical significance was set at *P* < 0.05. The receiver-operating characteristic curve (ROC curve) is a graphical representation that illustrates the diagnostic ability of a binary classification system with a varying discrimination threshold.

## Results

### Characteristics of the Patients

As shown in **Table 1**, a total of 68 patients were enrolled in this study, including 30 males (44.1%) and 38 females (55.9%), with a mean age of 48.3 ± 11.3 years. Six of them (8.8%) underwent lobectomy thyroidectomy+ CND, 2(2.9%) underwent lobectomy thyroidectomy + CND+ ipsilateral LND, 4(5.9%) underwent subtotal thyroidectomy+ CND, 9(13.2%) underwent subtotal thyroidectomy+ CND+ ipsilateral LND, 10(14.7%) underwent total thyroidectomy+ CND, 28(41.2%) underwent total thyroidectomy+ CND+ ipsilateral LND, 9(13.2%) underwent total thyroidectomy+ CND+ bilateral LND and postoperative pathology confirmed that 5 (7.4%) had capsule invasion. Lymph node metastasis occurred in 34 (50.0%), of whom 7 (10.3%) had central lymph node metastasis and 27 (39.7%) had lateral neck lymph node metastasis. After the follow-up period (median 79 months), 39 (57.4%) patients were classified as being in remission, 8 (11.8%) as stable, 21 (30.9%) as progressive, and five patients as recurrence.

**Table 1:** Baseline clinicopathological characteristics of MTC patients.

Features	N (%)	Features	N (%)
**Total**	68	Yes	3 (4.4%)
**Sex**		**Thyroid function**	
Female	38 (59.1%)	euthyroid	57 (83.8%)
Male	30 (44.1%)	hypothyroid	2 (2.9%)
**Age (years)**	48.3 ± 11.3	hyperthyroid	0 (0%)
**Largest tumor size (cm)**	1.2 (0.6-2.2)	subclinical hypothyroid	7 (10.3%)
**Multifocality**		subclinical hyperthyroid	2 (2.9%)
No	57 (83.2%)	**Type of operation**	
Yes	11 (16.2%)	Lobectomy thyroidectomy+ CND	6 (8.8%)
**Bilateral**		Lobectomy thyroidectomy+ CND+ Ipsilateral LND	2 (2.9%)
No	60 (88.2%)	Subtotal thyroidectomy+ CND	4 (5.9%)
Yes	8 (11.8%)	Subtotal thyroidectomy+ CND+ Ipsilateral LND	9 (13.2%)
**T stage**		Total thyroidectomy+ CND	10 (14.7%)
T1+T2	62 (91.2%)	Total thyroidectomy+ CND+ Ipsilateral LND	28 (41.2%)
T3+T4	6 (8.8%)	Total thyroidectomy+ CND+ Bilateral LND	9 (13.2%)
**N stage**		**Calcitonin changes in follow-up**	
N0	34 (50.0%)	Remission	39 (57.4%)
N1a	7 (10.3%)	Stable	8 (11.8%)
N1b	27 (39.7%)	Progress	21 (30.9%)
**TNM stage**		**Inflammation index**	
I+II	34 (50%)	LMR	5.2 ± 1.7
III+IV	34 (50%)	NLR	1.7 ± 0.6
**Capsule invasion**		PLR	115.1 ± 31.2
NO	63 (92.6%)	MPV	9 (8.1-10.0)
YES	5 (7.4%)	PDW	16.1 (15.5-16.5)
**Hashimoto’s thyroiditis**		**Follow up time (months)**	72.0 ± 32.4
No	65 (95.6%)		

CND, central neck dissection; LND, lateral neck dissection; LMR, lymphocyte-to-monocyte ratio; NLR, neutrophil-to-lymphocyte ratio; PLR, platelet-to-lymphocyte ratio; MPV, average platelet volume; PDW, platelet distribution width.

### Predictive Performance of Inflammatory Markers

**Figure 2:**
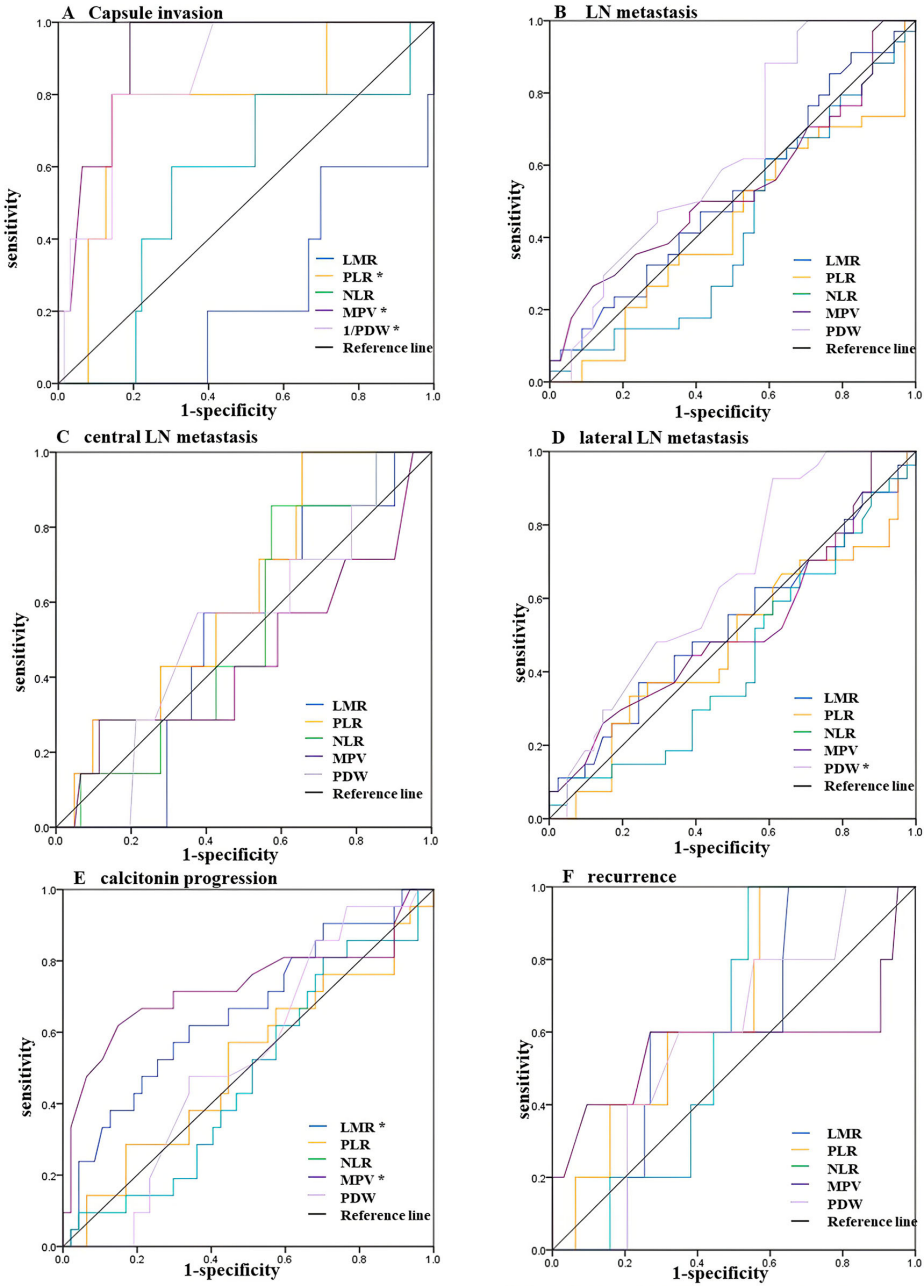
ROC curves for the preoperative LMR, PLR, NLR, MPV and PDW to predict **(A)** Capsule invasion, **(B)** LN metastasis, **(C)** central LN metastasis, **(D)** lateral LN metastasis, **(E)** calcitonin progression, and **(F)** recurrence in MTC patients. * Statistically significant *P-values* (*P* < 0.05). LMR, lymphocyte-to-monocyte ratio; NLR, neutrophil-to-lymphocyte ratio; PLR, platelet-to-lymphocyte ratio; MPV, average platelet volume; PDW, platelet distribution width; LN, lymph node; MTC, medullary thyroid carcinoma.

As shown in **Figure 2**, we used the ROC curve to evaluate the predictive power of each inflammatory index for capsule invasion, lymph node (LN) metastasis, postoperative Ctn progression, and recurrence. The corresponding AUC values are shown in **Table 2**. For predicting capsule invasion, the AUCs of PLR, PDW and MPV were 0.771 (95% CI: 0.546–0.996, *P*=0.045), 0.857 (95% CI: 0.726–0.988, *P*=0.008) and 0.914 (95% CI: 0.833–0.996, *P*=0.002), respectively. However, LMR and NLR showed relatively low discriminative power. We then checked the discriminative value of each inflammatory indices in LN metastases, central LN metastases, and lateral LN metastases and found that all inflammatory indices had no discriminative power for LN metastases or central LN metastases. However, for predicting lateral LN metastasis, PDW was found to have significant discriminatory power, with an AUC measurement of 0.645 (*P*=0.044), and a cut-off value of 16.4. Finally, we also examined the predictive performance of inflammatory indices for postoperative Ctn progression and recurrence, although none of them predicted recurrence. LMR and MPV were found to be significantly predictive of postoperative Ctn progression, with AUC values of 0.657 (*P*=0.040) and 0.728 (*P*=0.003), respectively. With an LMR cut-off value of 4.7, the sensitivity and specificity were 61.9% and 66.0%, respectively. With an MPV cut-off value of 8.2, specificity was 85.1%, and sensitivity was 61.9%.

**Table 2:** Area under the ROC curve.

Features	AUC	95%Cl	*P*-value	Cut-off	sensitivity	specificity
**Capsule invasion**						
LMR	0.749	0.540-0.958	0.065	/	/	/
PLR	0.771	0.546-0.996	0.045	138.3	0.800	0.857
NLR	0.562	0.307-0.816	0.647	/	/	/
MPV	0.914	0.833-0.996	0.002	10.0	1.000	0.810
PDW	0.857	0.726-0.988	0.008	14.5	0.800	0.857
**LN metastasis**						
LMR	0.523	0.385-0.661	0.745	/	/	/
PLR	0.433	0.295-0.571	0.342	/	/	/
NLR	0.423	0.285-0.562	0.278	/	/	/
MPV	0.535	0.396-0.675	0.615	/	/	/
PDW	0.629	0.495-0.763	0.067	/	/	/
**Central LN metastasis**						
LMR	0.506	0.324-0.688	0.96	/	/	/
PLR	0.616	0.425-0.807	0.318	/	/	/
NLR	0.527	0.333-0.721	0.816	/	/	/
MPV	0.452	0.198-0.706	0.679	/	/	/
PDW	0.527	0.319-0.735	0.816	/	/	/
**Lateral LN metastasis**						
LMR	0.526	0.382-0.671	0.716	/	/	/
PLR	0.475	0.329-0.620	0.726	/	/	/
NLR	0.430	0.290-0.570	0.334	/	/	/
MPV	0.519	0.374-0.663	0.797	/	/	/
PDW	0.645	0.516-0.775	0.044	16.4	0.926	0.390
**Calcitonin progress**						
LMR	0.657	0.513-0.801	0.040	4.7	0.619	0.660
PLR	0.511	0.357-0.664	0.889	/	/	/
NLR	0.474	0.328-0.620	0.735	/	/	/
MPV	0.728	0.574-0.881	0.003	8.2	0.619	0.851
PDW	0.528	0.389-0.668	0.710	/	/	/
**Recurrence**						
LMR	0.598	0.402-0.795	0.466	/	/	/
PLR	0.667	0.467-0.866	0.217	/	/	/
NLR	0.597	0.440-0.754	0.474	/	/	/
MPV	0.568	0.205-0.931	0.613	/	/	/
PDW	0.589	0.370-0.808	0.511	/	/	/

LMR, lymphocyte-to-monocyte ratio; NLR, neutrophil-to-lymphocyte ratio; PLR, platelet-to-lymphocyte ratio; MPV, average platelet volume; PDW, platelet distribution width.

For P ≤0.05, cut-off, sensitivity, and specificity are not shown.

### Logistic Regression Analysis of Risk Factors for Lateral LN Metastasis and Postoperative Ctn Progression

We performed univariate and multivariate analyzes to further illustrate the risk factors for lateral LN metastasis and postoperative progression of Ctn (**Table 3**). Univariate analysis revealed that bilateral tumors (*P*=0.011), multifocality (*P*=0.035), largest tumor size ≥1 cm (*P*=0.003) and PDW ≤16.4 (*P*=0.018) were significantly related to lateral LN metastasis; further multivariate regression analysis showed that PDW ≤16.4 and largest tumor size ≥1 cm were potential independent risk factors of lateral LN metastasis. Specifically, the risk of lateral LN metastasis was 7.8 times higher in patients with a PDW ≤ 16.4 than in patients with a PDW > 16.4 (OR=7.8, 95% CI: 1.532-39.720, *P*=0.013) and the risk of lateral LN metastasis in patients with the largest tumor size ≥1 cm was 4.8 times higher than in patients with the largest tumor size < 1 cm (OR=4.833, 95% CI: 1.514-15.427, *P*=0.008).

**Table 3:** Univariate and multivariate analyses of the risk factors for lateral LN metastasis.

Features	Non-N1b	N1b	Univariate *p*-value	Multivariate	
				OR(95%CI)	*p-*value
**Age (year)**			0.421		
≤55	18	31			
>55	9	10			
**Sex**			0.649		
female	16	22			
male	11	19			
**Bilateral**			0.011		
No	20	40			
Yes	7	1			
**Multifocality**			0.035		
No	19	38			
Yes	8	3			
**Capsule invasion**			>0.05		
No	25	38			
Yes	2	3			
**Largest tumor size (cm)**			0.003		
≤1	6	24		1	
>1	21	17		4.833 (1.514-15.427)	0.008
**PDW**			0.018		
PDW ≤ 16.4	2	13		7.8 (1.532-39.720)	0.013
PDW>16.4	25	28		1	

Similarly, we also examined the risk factors for postoperative Ctn progression (**Table 4**). Univariate analysis revealed that the largest tumor size ≥1 cm (*P*=0.024), LN metastasis (*P*=0.001), MPV ≤8.2 (*P* < 0.01), and LMR ≤4.7 (*P*=0.032) were significantly associated with postoperative Ctn progression; further multivariate analysis revealed that MPV ≤8.2, LMR ≤4.7, and N1 were potential independent risk factors for postoperative Ctn progression. Specifically, the risk of postoperative Ctn progression was 14.0 times higher in patients with MPV ≤8.2 than in patients with MPV > 8.2 (OR=13.999, 95% CI: 2.842-68.965, P=0.001), and it was 4.8 times higher than in patients with LMR > 4.7 (OR=4.790, 95% CI: 1.034-22.187, P =0.045), and in patients with LN metastases it was 45.9 times higher than in patients without metastases (OR=45.890, 95% CI: 3.879-542.936, P=0.002).

**Table 4:** Univariate and multivariate analyses of the risk factors for calcitonin progression.

Features	Non-progressive group	Progressive group	Univariate *p*-value	Multivariate	
				OR (95%CI)	*P*-value
**Age (year)**			0.938		
≤55	34	15			
>55	13	6			
**Sex**			0.359		
female	28	10			
male	19	11			
**Largest tumor size (cm)**			0.024		
≤1	25	5			
>1	22	16			
**Bilateral**			0.981		
No	42	18			
Yes	5	3			
**Multifocality**			0.942		
No	40	17			
Yes	7	4			
**Capsule invasion**			0.294		
No	42	21			
Yes	5	0			
**Coexisting thyroiditis**			1		
No	45	20			
Yes	2	1			
**T stage**			0.336		
I+II	45	18			
III+IV	2	3			
**N stage**			0.001		
N0	30	14		1	
N1	17	17		45.890(3.879-542.936)	0.002
**N stage**			0.049		
N0+N1a	32	9			
N1b	15	12			
**TNM stage**			0.001		
I+II	30	4			
III+IV	17	17			
**MPV**			<0.01		
>8.2	10	14		1	
≤8.2	37	7		13.999(2.842-68.965)	0.001
**LMR**	13/8	16/31	0.032		
>4.7	16	13		1	
≤4.7	31	8		4.790(1.034-22.187)	0.045

### Predictive Performance of MPV and LMR Together for Postoperative Ctn Progression

**Figure 3:**
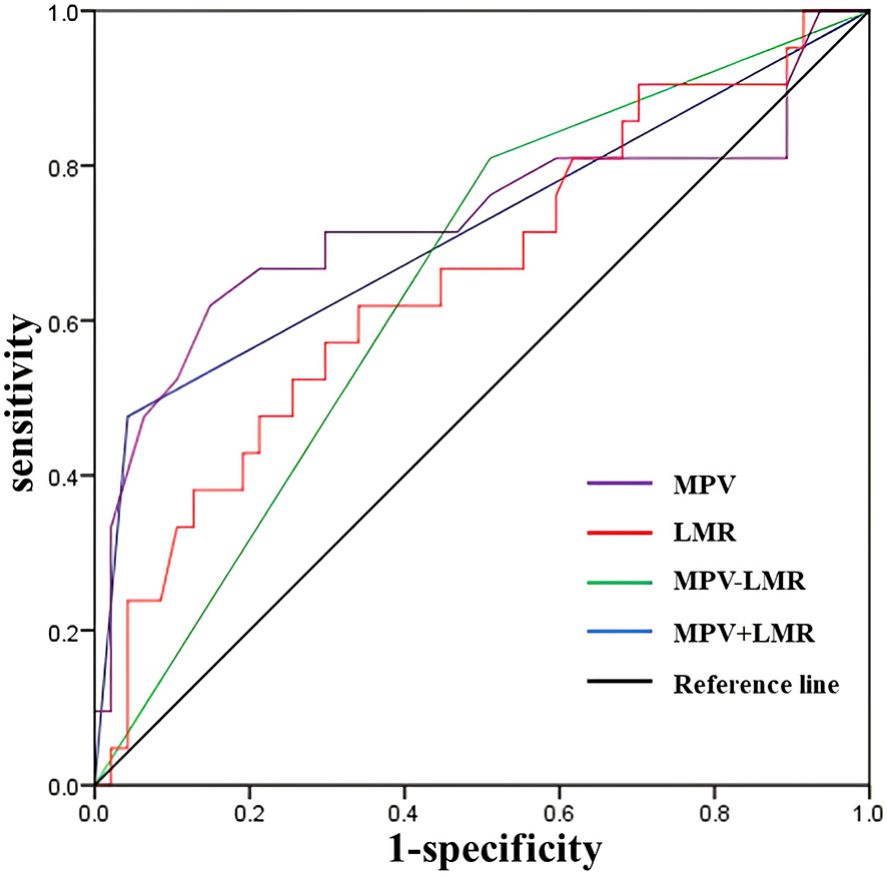
ROC curves for the combination of preoperative MPV and LMR to predict calcitonin progression. LMR, lymphocyte-to-monocyte ratio; MPV, mean platelet volume. MPV-LMR, MPV ≤8.2 or LMR ≤4.7; MPV+LMR, MPV ≤8.2 and LMR ≤4.7.

Based on the above, we further examined the predictive power of the combination of MPV and LMR for postoperative Ctn progression. As shown in **Figure 3** and **Table 5**, according to the cut-off value obtained from the ROC curve, we defined either MPV ≤8.2 or LMR ≤4.7 as MPV-LMR and MPV ≤8.2 and LMR ≤4.7 simultaneously as MPV+LMR simultaneously. The sensitivity of MPV-LMR in predicting postoperative Ctn progression increased from 61.9% to 81%, while the specificity of MPV+LMR in predicting postoperative Ctn progression increased from 66% to 95.7%.

**Table 5:** Area under the ROC curve.

	AUC	95%Cl	*P*-value	Cut-off	Sensitivity	Specificity
MPV	0.728	0.574-0.881	0.003	8.2	0.619	0.851
LMR	0.657	0.513-0.801	0.040	4.7	0.619	0.660
MPV-LMR	0.649	0.512-0.786	0.050	0.5	0.810	0.489
MPV**+**LMR	0.717	0.570-0.864	0.050	0.5	0.476	0.957

MPV-LMR, MPV ≤ 8.2 or LMR ≤ 4.7; MPV+LMR, MPV ≤ 8.2 and LMR ≤ 4.7.

LMR, lymphocyte-to-monocyte ratio; MPV, mean platelet volume.

## Discussion

The inflammatory response in the tumor microenvironment is involved in the dual process of tumor progression and suppression (9). Inflammatory indices are newly discovered tumor markers based on host inflammatory response. Studies have shown that they correlate with clinicopathological features and prognosis in pancreatic cancer, lung cancer, and anaplastic thyroid carcinoma (2, 4, 10). However, there are few reports on MTC. Therefore, in our study, we sought to clarify and compare the applications of inflammatory indices as MTC tumor markers. In addition, we investigated the relationship between inflammatory indices and postoperative Ctn progression for the first time.

The development of a scored-inflammatory marker system established here is useful to predict the invasiveness and progress of MTC patients. The inflammatory models established in this study can be used to predict prognosis.

The ROC analysis showed that PDW was significantly associated with lateral LN metastasis, preoperative PLR, PDW, and MPV were predictive of invasion, and MPV and LMR were predictive of postoperative Ctn progression. Further multivariate analysis revealed PDW ≤16.4 was a potential independent factor for predicting lateral LN metastasis, and LMR ≤4.7 and MPV ≤8.2 were potential independent factors for predicting postoperative Ctn progression. Notably, the combination of LMR and MPV has improved sensitivity and specificity for predicting postoperative Ctn progression.

Previous studies investigated the relationship between tumor invasion and inflammatory indices, but their conclusions were conflicting. After a retrospective analysis of data from 205 patients with papillary thyroid carcinoma (PTC) in Greece, Manatakis et al. found that the NLR was significantly higher in carcinomas with extrathyroidal invasion (2.74 ± 1.24 versus 2.39 ± 0.96, *P*=0 04) (11), but other studies reached different conclusions (12–16). Also in our study, invasion was found to be not significantly associated with preoperative NLR but significantly associated with high PLR, MPV and low PDW. The specific mechanism is not clear, although it may be possible that the release of various inflammatory mediators induces an increase in platelets, while activated platelets contribute to the release of platelet-derived growth factor, platelet-activating factor, and vascular endothelial growth factor, which accelerate tumor-related blood vessel formation and extracellular matrix degradation, promoting tumor growth and distant metastasis (17). Although lymphocytes are the smallest white blood cells, they can induce the production of various cytotoxins, such as perforin, which directly or indirectly exert anti-tumoral effects (18). To some extent, lymphopenia indicates a weakened antitumoral immune response, which lead to more invasion and poorer prognosis (19). Therefore, PLR could be a potential tumor marker for predicting invasion in MTC.

However, some studies have indicated that platelet activation is related to platelet size rather than platelet number (20). MPV and PDW are common indicators of platelet volume. MPV indicates the mean platelet size and reflects the platelet production rate and stimulation (21). Larger platelets have stronger metabolic and enzymatic activities than smaller platelets (22). After reviewing data from 45 patients with gastric cancer and 20 healthy controls, Osada et al. found that MPV was higher in patients with gastric cancer than in healthy controls (23). However, Inagaki et al. found that the MPV of patients with advanced non-small cell lung cancer was lower than that of the control group (24). Similarly, our study suggests that a high MPV was predictive of invasion (AUC=0.914, 95% CI: 0.833–0.996, *P*=0.002), but an MPV ≤8.2 (OR=13.999, 95% CI: 2.842–68.965, *P*=0.001) was a potential independent risk factor for postoperative Ctn progression. This seemingly contradictory phenomenon highlights the complicated interactions between inflammatory factors in the tumor microenvironment. Moreover, it could also be explained by the indolent nature of DTC.

The PDW reflects the uniformity of platelet volume, and higher values indicate that abnormally small and large platelets are in circulation (25). Compared with MPV, PDW is more reliable in evaluating the causes of thrombocytopenia (26). Cheng et al. found a lower PDW in gastric cancer patients compared with the healthy control group and that a PDW ≤ 11.95 was an independent risk factor for disease-free survival of T1 gastric cancer (27). Similarly, we found that PDW had predictive power for invasion and lateral LN metastasis, and that PDW ≤16.4 (OR=0.128; 95% CI: 0.025–0.653; *P*=0.013) was a potential independent risk factor for lateral LN metastasis. This suggests that PDW is a potential tumor marker for MTC, and the lower its value, the worse the clinicopathological features of the tumor.

Moreover, our study was the first to find that an LMR ≤4.7 (OR=4.790; 95% CI: 1.034–22.187; *P*=0.045) was a potential independent risk factor for postoperative Ctn progression. Similarly, some studies have indicated that low LMR is associated with poor prognosis of PTC and anaplastic thyroid carcinoma (4, 28). This could be explained by the fact that circulating monocytes can transform into myeloid suppressor cells and tumor-associated macrophages, which are involved in tumor growth, invasion, and metastasis by accelerating the epithelial-mesenchymal transition (29, 30). Finally, we investigated the influence of the appropriate combination of inflammatory indices on diagnostic efficiency. The analysis showed that the sensitivity of MPV-LMR in predicting postoperative Ctn progression increased from 61.9% to 81%, while the specificity of MPV+LMR in predicting postoperative Ctn progression increased from 66% to 95.7%.

Limited by the small number of patients with recurrence (n=5), no significant association between LMR and recurrence was found in our study, but 5 patients were classified as postoperative Ctn progression, and some previous studies found that postoperative Ctn is significantly related to MTC prognosis (31), suggesting that LMR has potential for evaluating MTC prognosis.

Our study has several limitations. First, this was a retrospective analysis. Second, although patients with diseases that could influence complete blood count were excluded, there may have been some unknown or undetectable factors that could potentially influence our results. Finally, the number of cases included in our study was relatively small. Further large-scale and prospective studies are needed to confirm our conclusions.

## Conclusions

In conclusion, this study preliminarily explored the potential applications of inflammatory indices in the clinicopathological features and prognosis of MTC. We found that inflammatory indices were associated with the clinicopathological characteristics and postoperative Ctn progression in MTC. Specifically, PDW was predictive of lymph node metastasis, PLR, PDW, and MPV were predictive of capsule invasion, and MPV and LMR were predictive of postoperative Ctn progression. Remarkably, the appropriate combination of MPV and LMR could improve the specificity and sensitivity of predicting postoperative Ctn progression. Overall, this suggests that inflammatory indices are potential biomarkers for predicting the clinicopathological features and prognosis of medullary thyroid carcinoma.

## Data Availability Statement

The raw data supporting the conclusions of this article will be made available by the authors, without undue reservation.

## Ethics Statement

The studies involving human participants were reviewed and approved by China-Japan Union Hospital Institutional Review Board. Written informed consent for participation was not required for this study in accordance with the national legislation and the institutional requirements. Written informed consent was obtained from the individual(s) for the publication of any potentially identifiable images or data included in this article.

## Author Contributions

HS, CL, and NL contributed to the study conception and design. Material preparation and data collection were carried out by CL, HZ, SL, DZ, and JL. All authors proposed many professional suggestions when data analysis. The first draft of the manuscript was written by CL. GD and NL contributed to manuscript review and editing. HS was responsible for project administration and supervision. All authors contributed to the article and approved the submitted version.

## Funding

This study was supported by the National Nature Science Foundation of China [81972499]; Jilin Province educational program [JJKH20211142KJ], the Jilin Province Science and Technology Development Program [20210402011GH]; the Program of Jilin Provincial Finance Department [2020SCZ03]; Jilin University Bethune Project [2020B14].

## Conflict of Interest

The authors declare that the research was conducted in the absence of any commercial or financial relationships that could be construed as a potential conflict of interest.

## Publisher’s Note

All claims expressed in this article are solely those of the authors and do not necessarily represent those of their affiliated organizations, or those of the publisher, the editors and the reviewers. Any product that may be evaluated in this article, or claim that may be made by its manufacturer, is not guaranteed or endorsed by the publisher.

## Supplementary Material

Supplementary Table 1 Classification based on postoperative calcitonin.
