# Supplementary material for: Prognostic Impact of Inflammatory Markers PLR, LMR, PDW, MPV in Medullary Thyroid Carcinoma
Source: Front Endocrinol (Lausanne). 2022 Mar 8;13:861869. doi: 10.3389/fendo.2022.861869 (PMC8957807; doi:10.3389/fendo.2022.861869)
Supplement: Supplementary Table 1 — Classification based on postoperative calcitonin. [file Table_1.docx]

**Supplementary table 1: Classification based on postoperative calcitonin**

| **Groups** | **Definition** |
| --- | --- |
| Remission | Postoperative Ctn levels decreased to normal levels and remained steady |
| Stable | Postoperative Ctn levels were stable but did not decrease to normal levels |
| Progression | Postoperative Ctn level increased to 150 pg/mL or the doubling time was <12 months |
